# CrossFit-Induced Rhabdomyolysis in a Brazilian Coach: A Case Report

**DOI:** 10.7759/cureus.64015

**Published:** 2024-07-07

**Authors:** Leonardo Busanello Mata Alves, Eduardo Campos Martins, Luiz Fernando Sartori Centenaro

**Affiliations:** 1 Surgery, Federal University of Santa Catarina, Florianópolis, BRA; 2 Hip Surgery, Fractures Institute, Baía Sul Medical Center, Florianópolis, BRA; 3 Shoulder Surgery, Fractures Institute, Baía Sul Medical Center, Florianópolis, BRA

**Keywords:** sports, pain, injuries, emergency, rhabdomyolysis

## Abstract

CrossFit is a high-intensity physical activity modality that, despite its numerous health benefits, poses a risk of exercise-related injuries. The rare but serious complication of exertional rhabdomyolysis is a good example. There are few case reports describing this condition, and to the best of the author's knowledge, this is the first published case of CrossFit-induced rhabdomyolysis reported in Brazil - one of the most influential countries in the world of CrossFit. Our case report describes a 45-year-old male, an experienced Level 2 CrossFit Coach, who presented with progressive upper limb pain and dark urine two days after a routine CrossFit workout. Physical examination revealed muscle stiffening and pain upon palpation. Laboratory tests showed significantly elevated creatine phosphokinase (CPK) levels (126.891 U/L) and abnormal values of lactate dehydrogenase, alanine aminotransferase, and aspartate aminotransferase. The patient was diagnosed with exercise-induced rhabdomyolysis and treated with aggressive intravenous and oral hydration, with complete clinical improvement by the fifth day of hospitalization. The patient was dismissed without any complications and with progressively decreasing levels of CPK, with ambulatorial follow-up arranged. CrossFit-induced rhabdomyolysis, although rare, represents an important health concern due to the possibility of severe systemic consequences. The present case highlights the importance of early detection and treatment of exertional rhabdomyolysis, even in well-conditioned athletes.

## Introduction

CrossFit is an increasingly popular physical activity program that was created in 2000, with currently over 15,000 affiliated training centers and 15 million athletes [1]. It is based on sets of functional movements performed at high intensity. It has proven benefits for overall health, such as improved cardiovascular fitness, increased strength, and enhanced flexibility, as well as promoting the holistic development of fine motor skills and coordination [1]. However, like other high-intensity, high-impact sports and physical activities, CrossFit may be associated with an increased risk of exercise-related injuries [2,3].

Among the various injuries associated with CrossFit described in the literature, exertional rhabdomyolysis is a rare but serious complication [4]. Due to the limited number of published case reports, it may be underdiagnosed and underreported [5-9]. This condition occurs due to the leakage of intracellular contents from muscle cells into the extracellular environment. It can be precipitated by musculoskeletal trauma, rupture of muscle fibers, or muscular necrosis. Clinically, it manifests with both localized symptoms, such as intense myalgia and weakness, and systemic manifestations, including malaise, fever, myoglobinuria, hyperkalemia, and acute kidney injury [10,11].

Thus, exertional rhabdomyolysis represents a significant health risk, owing to its potential to cause severe systemic complications. Our case report describes a 45-year-old male with extensive experience in CrossFit training who presented with the typical clinical features of exertional rhabdomyolysis.

## Case presentation

A 45-year-old Caucasian male, a Level 2 CrossFit Coach (CF-L2), presented to the Emergency Department with complaints of progressive pain in his upper limbs and the sudden onset of dark urine. The patient denied having any significant past medical history or long-term medication use. He also denied local trauma but reported that two days prior to the onset of symptoms, he participated in a CrossFit workout, which included a two-mile run, 200 push-ups, 100 pull-ups, and 300 air squats. The workout was within his usual standards of physical exertion, without excessive fatigue during the session, and the hydration during the workout was adequate. However, he was forced to stop halfway through due to sudden discomfort in his upper limbs. The symptoms progressed over the following days, worsening despite the use of low doses of acetaminophen and rest.

Upon initial assessment, physical examination revealed bilateral muscle hardening in the upper limbs, with pain during palpation and passive movements. His vital signs were within normal limits. Initial laboratory assessment showed creatine phosphokinase (CPK) of 126.891 U/L, aspartate aminotransferase (AST) of 2.154 U/L, alanine aminotransferase (ALT) of 568 U/L, and lactate dehydrogenase (LDH) of 4.067 U/L. However, renal function was preserved with serum creatinine, serum potassium, and blood urea nitrogen within normal limits (as shown in Table 1).

**Table 1:** Laboratory tests findings on the admission day, day five from admission, and day 25 from admission

	Reference Values	Admission Day	Day 5	Follow-Up (Day 25 After Admission)
Creatine Phosphokinase (U/L)	33-211	126.891	5.467	286
Creatinine (mg/dl)	0.6-1.10	1.1	1.2	1.1
Urea (mg/dl)	10-50	35	34	32
Potassium (mEq/L)	3.5-4.5	4.0	4.3	-
Calcium (mg/dl)	8.6-10.3	8.6	-	-
Aspartate aminotransferase (U/L)	5-40	2.154	-	35
Alanine aminotransferase (U/L)	10-49	568	-	28
Lactate dehydrogenase (U/L)	120-246	4.067	-	240
Urinalysis				
Color	Yellow	Dark yellow	-	-
pH	05-06	6.5	-	-
Density	1.016-1.025	1.024	-	-
Hemoglobin	Negative	Positive (+++)	-	-
Nitrite	Negative	Negative	-	-
Leukocytes	Negative	Negative	-	-
Bilirubin	Negative	Negative	-	-
Bacteria	Negative	Negative	-	-
Crystal	Negative	Negative	-	-
Ketone	Negative	Negative	-	-

**Figure 1:**
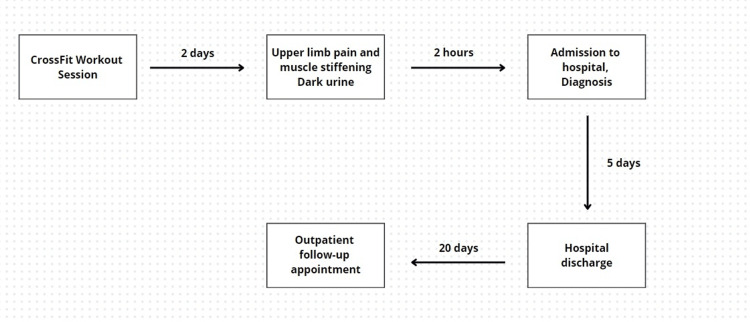
Sequence of events regarding the presented clinical case

Given the clinical and laboratory findings, along with the history of strenuous exercise, the patient was hospitalized for clinical management of exercise-induced rhabdomyolysis. He was treated with analgesia, vigorous intravenous hydration, and oral hydration over five days, with monitoring of CPK levels. On the second day of hospitalization, he sustained a urine output of 2.5 mL/kg/h, with normalization of urine color and a significant decrease in CPK levels. He experienced no complications during his hospital stay, maintaining normal renal function. By the fifth day, he showed complete clinical improvement and a substantial reduction in CPK levels and other biomarkers. The patient was discharged with arrangements for outpatient follow-up. After 20 days of discharge, the patient did not report any new symptoms and had a persistent mild elevation of CPK levels, with complete normalization of other serum biomarkers. Figure 1 displays a flowchart depicting the chronology of events regarding the clinical case.

## Discussion

CrossFit combines high-intensity exercises performed over short periods with minimal rest intervals. It is based on the repeated execution of physical activities such as running, weightlifting, and bodyweight exercises. The sport has been increasing in popularity worldwide, with the number of practitioners estimated to be between two and four million [2,3]. Among the practitioners who want to go deeper into the practices of the sport, there are also four levels of individual certification as a CrossFit Coach, from CF1 to CF4 [12]. However, the cost-benefit ratio of CrossFit should be considered, as the average injury rate associated with the training program is approximately 19% [3].

The incidence of exercise-related rhabdomyolysis due to high-intensity exercise is known to be low, being estimated at 29.9 cases per 100,000 individuals, with only a few case reports linking the condition to the practice of CrossFit [3,4-8]. In these cases, rhabdomyolysis can result from a combination of prolonged high-intensity exercise, strenuous training outside of usual standards, medication use, direct trauma, or metabolic diseases. It results in muscle necrosis, with the release of intracellular enzymes in large amounts causing the clinical presentation of the disease [10,11].

A study conducted by Hopkins et al. analyzed 11 admitted patients with a diagnosis of CrossFit-induced rhabdomyolysis of 523 patients with injuries after CrossFit practice, describing risk factors, clinical presentation, and prognosis related to the clinical condition [9]. In accordance with the findings described in the study, the patient in the case was a male (81.8%) of 45 years (34.9±9.7 years) who presented a clinical picture of darkened urine (90.9%) and pain in the upper limbs (54.5%) with two days of duration of symptoms (2.9±1.5 days). Also, more than 72% of the study patients admitted to the emergency department had CPK values above 20,000 IU/L. However, the study noted that 54.5% of cases occurred in CrossFit beginners, while our patient was already an advanced practitioner, certified as a Level 2 Coach. Such certification indicates that besides being an avid practitioner of the sport, the patient has in-depth knowledge about the mechanics of movements and is also able to lead CrossFit classes.

Thus, when suspecting exertional rhabdomyolysis, it is essential to conduct a comprehensive history, a complete physical examination, and request additional laboratory tests. Both the history and the focused physical examination can demonstrate the clinical triad of rhabdomyolysis, which consists of myalgia, muscle weakness, and myoglobinuria. However, although classic, this is absent in more than 50% of the cases, and the condition may present only with non-specific clinical signs such as fever, malaise, nausea, muscular edema, and palpitations [11]. Also, a history of strenuous exercise without adequate water intake should raise suspicion of the disease, as proper hydration is proven to prevent heat-induced disorders such as exertional rhabdomyolysis [13].

Therefore, laboratory investigation is essential, with the diagnosis defined by an elevation of serum CPK above three to five times the upper limit of normality [13,14]. Other laboratory abnormalities may be present due to intense intracellular content leakage, such as increased values of AST, ALT, LDH, serum potassium, and serum phosphate, with low values of serum calcium [10,11]. The main finding of urinalysis is the presence of myoglobin, typically detected only when it exceeds 0.3 mg/L [10]. The sensitivity and specificity of this finding are widely scattered and vary significantly among studies [11]. Other nonspecific findings on urinalysis include decreased urinary pH and the presence of proteinuria [10,11].

It is important to highlight that exhausting physical exercise can cause an increase in CPK values by up to 30 times the value of normality without any clinical manifestation [9,15]. This isolated elevation can be portrayed as a confounding factor, therefore a high index of suspicion for exertional rhabdomyolysis should be aimed at patients with corresponding history, risk factors, clinical picture, and laboratory findings.

In rhabdomyolysis, CPK values as low as 5.000 U/L may be sufficient to trigger severe complications, such as acute renal failure, severe electrolytic disorders, or even multiple organ failure [16]. Thus, it is of the utmost importance to have an early diagnosis and an immediate start to treatment to avoid such complications. With regard to treatment, there is no unique guideline for rhabdomyolysis, but intensive volume replacement with isotonic solutions, such as 0.9% sodium chloride, is conventionally initiated to maintain renal perfusion and optimize myoglobin clearance. The main objective is to maintain an elevated urine output, aiming for values between 200 and 300 mL/h (approximately 2-3 mL/kg/h) [7]. Due to potential complications, serial complementary tests should be conducted to evaluate renal function and electrolyte disturbances. Our patient received two liters of intravenous solution upon admission, along with high oral water intake, maintaining a high urine output in the first days of hospitalization.

Our study presented some non-negligible limitations. First, the daily measurement of the laboratory tests during the hospital stay was not carried out, with a treatment regimen guided mainly by the clinical improvement of the patient. Furthermore, values of serum calcium, serum potassium, ALT, AST, and LDH were not reassessed before hospital discharge. Finally, our patient was released with persistent elevated CPK levels (5.467 U/L), based on the possibility of close follow-up in an outpatient environment and the lack of symptoms or complications.

## Conclusions

CrossFit-induced rhabdomyolysis is a potentially serious condition with a high risk of morbidity and mortality, even in individuals with a high level of physical fitness. The small number of cases indicates that this is probably an underreported condition, requiring further research to determine its true incidence and to highlight relevant associated risk factors. For these reasons, health professionals should be aware of the diagnosis, thus facilitating early recognition of the condition and the establishment of adequate treatment required to avoid catastrophic sequelae.
